# Individualized intervention for frail non-dialysis elderly patients with chronic kidney disease: protocol for a randomized controlled trial

**DOI:** 10.1186/s12877-020-1491-6

**Published:** 2020-05-04

**Authors:** Jing Chang, Yun Gao, Xiang-Yang Fang, Su-Mei Zhao, Yuan-Ping Hou, Qian-Mei Sun

**Affiliations:** 1 Department of Internal Medicine, Beijing Chao-Yang Hospital, Capital Medical University, Gong-Ti South Road 8#, Chao-Yang District, Beijing, China; 2 Department of Nephrology, Beijing Chao-Yang Hospital, Capital Medical University, Beijing, China

**Keywords:** Frailty, Chronic kidney disease, Non-dialysis patients, Individualized intervention

## Abstract

**Background:**

Frailty describes an age-related clinical state and can be regarded as a predictive factor for fall, disability, hospitalization, and death in the elderly. Previous studies proved that frailty could be reversed or attenuated by multi-disciplinary intervention. However, only a few studies have been performed in non-dialysis patients with chronic kidney disease.

**Methods:**

A randomized parallel controlled trial will be conducted to compare an individualized intervention according to the consequence of the comprehensive geriatric assessment with routine treatment. A total of 242 individuals aged ≥65 years, who fulfill the Fried Phenotype of frailty and have chronic kidney disease stage 3–5 without dialysis will be recruited from the Department of Nephrology and Department of Internal Medicine, Beijing Chaoyang Hospital, Capital Medical University. The participants will be followed-up for 30 days and 12 months.

**Discussion:**

This protocol would be established to examine the efficiency of targeted intervention for frailty. If a positive consequence could be obtained, a novel treatment for frail elderly patients with chronic kidney disease who have never undergone dialysis can be carried out in routine clinical practice.

**Trial registration:**

The trial was prospectively registered at the Chinese Clinical Trials Registry with the registration number ChiCTR-IOR-17013429 on November 17, 2017.

## Background

The accelerated speed of aging and the heterogeneity of elderly have led to an increasing focus on the frailty, which is recognized as an age-associated state of increased vulnerability to external stressors resulting from the decreased physiological reserve function of multiple systems [1, 2]. As a multidimensional concept involving physical, psychological, social, and environmental aspects [3], frailty is consistently believed to be associated with falls, disability, hospitalization, institutionalization, mortality, and other poor outcomes [4]. Although frailty is closely related to aging, it is not tantamount to aging and can be regarded as unsuccessful aging [5]. Occasionally, distinguishing frailty from comorbidity and disability is challenging; however, comorbidity is an etiological risk factor for frailty, while disability is an outcome of frailty [6] as postulated by gerontologists.

The knowledge about frailty aided in the development of various evaluation tools. Nevertheless, there is no consensus on the tools for measuring frailty. The most commonly used instrument is Frailty phenotype proposed by Fried et al. in 2001 [7]. Frailty is a common and pervasive condition among the elderly. According to the results of several studies, the prevalence of frailty in the community of elderly is up to 7–10% [8]. Notably, frailty increases with age, and females have a more pronounced statistical prevalence than males [8]. Despite the prevalence of frailty and its association with negative outcomes, it can be easily overlooked owing to the lack of the corresponding chief complaint or the manifestations encompassed by comorbidity [9]. Consequently, individuals ≥65 years should be routinely screened for frailty.

Several direct or indirect factors might be involved in the development of frailty, including low education level, depression, malnutrition, polypharmacy, low physical activity, and concomitant chronic disease [10]. The prevalence of comorbidity in frail individuals is higher than in those without frailty [4], among which, chronic kidney disease (CKD) is one of the most common diseases closely linked to frailty. Some studies found that the prevalence of frailty among patients with CKD or end-stage renal disBGR01491ease (ESRD) is 3-fold than that of patients without the disease [6]. Furthermore, frail patients undergoing hemodialysis were associated with a 2.6-fold higher risk of mortality and 1.43-fold higher risk of hospitalizations, independent of age, sex, comorbidity, and disability as compared to patients without frailty [11]. Among patients treated with peritoneal dialysis, the frail patient has a significantly high rate of death or hospitalization, as well as, prolonged duration of stay per admission [12]. Moreover, no significant difference was detected in frailty between patients with hemodialysis and peritoneal dialysis [13]. Similarly, after adjusting the recipient, donor, and other confounders, the risk of early hospital readmission and mortality of frailty patients after kidney transplantation were 1.61-fold and 2.17-fold, respectively, more than those who were non-frail [14, 15].

Several studies demonstrated that frailty could be reversed or attenuated by nutritional supplementation, cognitive training, psychological intervention, or physical exercise, and multi-domain interventions are superior to monodomain interventions, among which, exercise training plays an irreplaceable role [16]. Some investigators speculated that exercise training could ameliorate poor physical functioning and frailty. Moreover, the survival rate s in patients with CKD can be improved [17]. In the case of frail patients undergoing CKD, most investigations focus on those treated with dialysis. Currently, only a few studies have been performed in frail non-dialysis patients. Thus, the objectives of this clinical study are 1) to explore the risk factors related to frailty in elderly CKD patients without dialysis, 2) to investigate the effect of individualized interventions of frailty on the prognosis of elderly patients with CKD who did not undergo dialysis.

## Methods

### Design outline

**Fig. 1:**
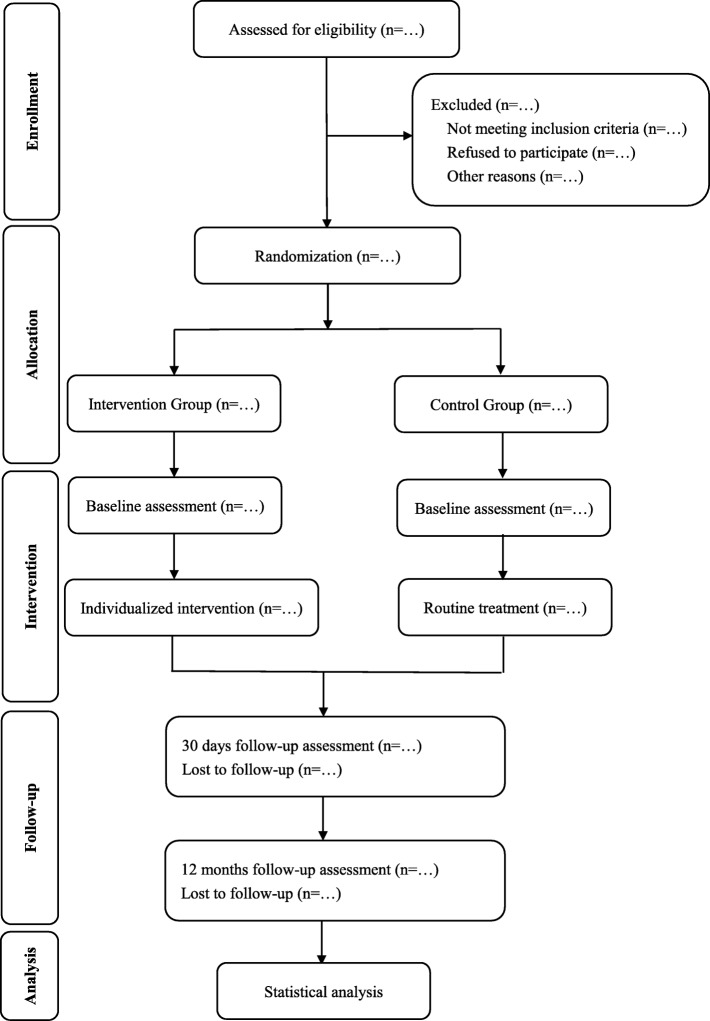
Schematic representation of the randomized controlled trial


A single center randomized parallel controlled trial with a follow-up period of 30 days and 12 months will be conducted. According to our pretest data, the reversion of frailty approached 25% in intervention group while 10% in control group, a total of 97 frail subjects are needed in each group (*α* = 0.05; *β* = 0.2; two-sided contrast). Considered a lost to follow-up rate of 20%, increased the sample size to 242. And, referring to relevant literatures, the frailty was reversed by individualized intervention, ratio is approaching 35 and 15% for the frailty versus non-frailty [16]. 71 patients for per group will be needed (*α* = 0.05; *β* = 0.2; two-sided contrast). Taking into account a rate of 20% loss to follow-up, increased the sample size to 178. Finally, the whole sample size is 242 in order to get the higher statistical power. All eligible participants will be randomized (1:1 allocation ratio) into control and intervention groups, and the latter would receive individualized interventions according to the consequence of the comprehensive geriatric assessment (CGA). The outcome data would be collected at baseline, 30 days, and 12 months (Fig. 1, Table 1).

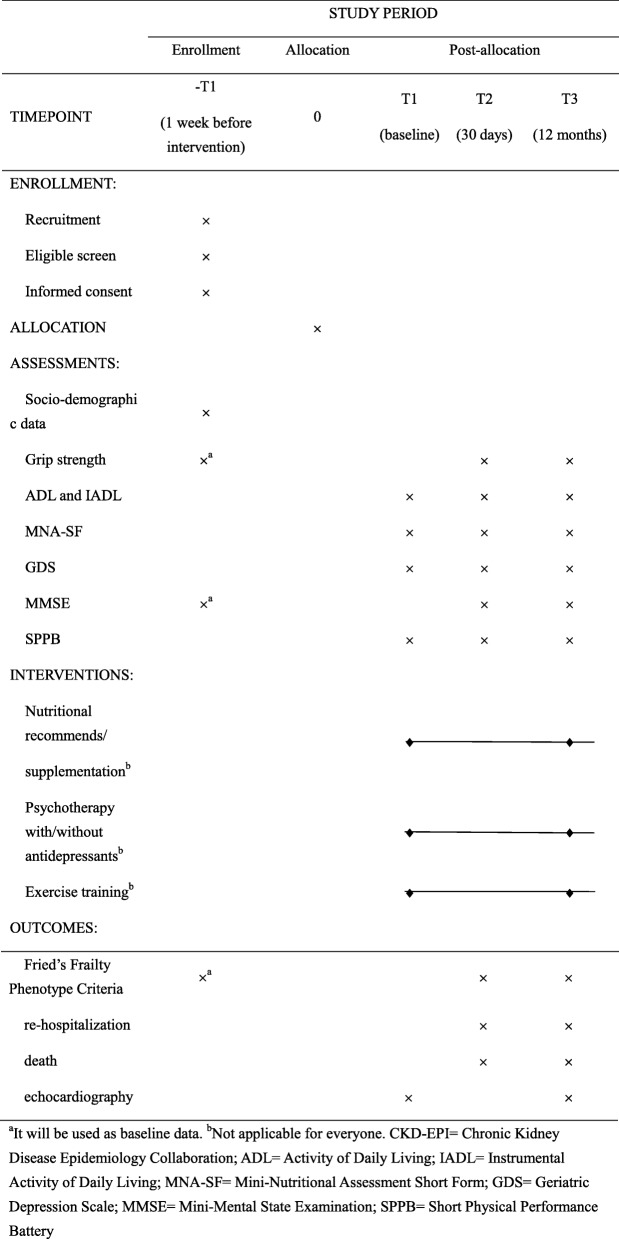

**Table 1:**
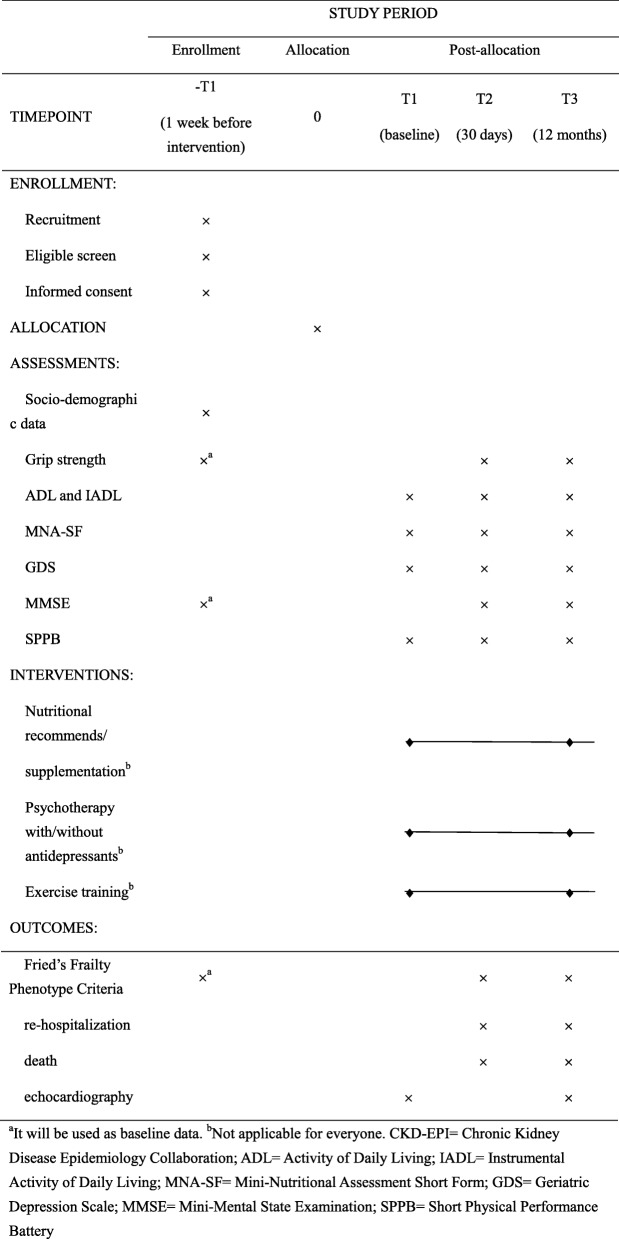
Schedule of the study

^a^It will be used as baseline data. ^b^Not applicable for everyone. *CKD-EPI* Chronic Kidney Disease Epidemiology Collaboration, *ADL* Activity of Daily Living, *IADL* Instrumental Activity of Daily Living, *MNA-SF* Mini-Nutritional Assessment Short Form, *GDS* Geriatric Depression Scale, *MMSE* Mini-Mental State Examination, *SPPB* Short Physical Performance Battery

The clinical study was supported by the Beijing Municipal Science and Technology Commission and the study protocol (Z171100000417055 V3.0) was reviewed and approved by the Medical Ethics Committee of Beijing Chaoyang Hospital, Capital Medical University, Beijing, China, and was approved unanimously (No. 2017-science-190). Participants will join in the clinical trial only after reading and signing the consent form. The right of participants to withdraw will be respected at any stage of the clinical trial. Deciding not to participate in the study will not affect usual medical care. Any collected data will be kept confidential. This study was registered as a randomized controlled clinical trial on 17 November 2017 (ChiCTR-IOR-17013429). Any change of the study scheme will be reported to the Medical Ethics Committee of Beijing Chaoyang Hospital, Capital Medical University and will be modified in the Chinese Clinical Trials Registry.

### Recruitment and eligibility

Eligible subjects will be identified and recruited in the Department of Nephrology and Department of Internal Medicine Beijing Chaoyang Hospital, Capital Medical University, including outpatients and inpatients. Recruitment will be conducted through printed materials (leaflets and posters). Patients will be required to fulfill the following criteria to qualify as study participants: 1) ≥65-years-old, 2) diagnosed as CKD stage 3–5, 3) never undergone hemodialysis or peritoneal dialysis, and 4) recognized as frailty by Fried scale. Chronic Kidney Disease Epidemiology Collaboration (CKD-EPI) formula is selected to estimate the glomerular filtration rate (eGFR); eGFR< 60 mL/min/1.73 m^2^ is defined as CKD stage 3–5 [18]. The informed consents will be signed after the eligible patients agree to participate in the investigation and allow access to their data during the follow-up either by clinic or telephone contact. Patients who are 1) undergoing dialysis, 2) considered with life expectancy < 1 year such as advanced cancer patients, 3) difficult to communicate with such as patients with severe cognitive impairment (Mini Mental State Examination score ≤ 17) [19]; or severe hearing disorder will be excluded from the study.

### Measurement and procedure

Frailty will be identified using Fried [20], including 5 components: 1) Shrinking, unintentional weight loss ≥10 pounds in last years or ≥ 5% of body weight in the previous year. 2) Weakness, based on grip strength, stratified by gender and body mass index (BMI) quartiles. For men, BMI ≤24 kg/m^2^, grip strength ≤29 kg; BMI 24.1–26 kg/m^2^, grip strength ≤30 kg; BMI 26.1–28 kg/m^2^, grip strength ≤30 kg; BMI > 28 kg/m^2^, grip strength ≤32 kg. For women, BMI ≤23 kg/m^2^, grip strength ≤17 kg; BMI 23.1–26 kg/m^2^, grip strength ≤17.3 kg; BMI 26.1–29 kg/m^2^, grip strength ≤18 kg; BMI > 29 kg/m^2^, grip strength ≤21 kg. In this study, we will measure the grip strength of the dominant hand twice to obtain the average. 3) Exhaustion, self-reported fatigue ≥3 days in the last week. 4) Low activity level, energy expenditure of physical activity per week < 383 kcal for men or < 270 kcal for women. 5) Slow gait speed, based on the time required for walking 15 ft. Walk time ≥ 7 s for men with ≤173 cm height or women with ≤159 cm height or walk time ≥ 6 s for men whose height was > 173 cm or women whose height was > 159 cm was considered as slowness. The presence of ≥3 criteria is defined as frailty, 1 or 2 is classified as pre-frailty, and 0 is regarded as non-frailty.

Frail elderly participants identified from candidates by Fried scale will receive CGA, containing activity of daily living (ADL) and instrumental activity of daily living (IADL) [21], mini-nutritional assessment short form (MNA-SF) [22], geriatric depression scale (GDS) [23], mini-mental state examination (MMSE) [19], and short physical performance battery (SPPB) [24]. ADL and IADL are designed to evaluate the basic self-care and necessary activities to run a household. The former includes eating, dressing, toileting, bathing, walking, and defecating, while the latter includes cooking, washing clothes, shopping, calling up, riding transportation, doing housework, managing money, and taking medicines. The dependence on performing ADL or IADL or both will be considered a disability. MNA-SF is a screening instrument to assess the nutrition status comprising of food intake, weight loss, mobility, disease acuity, BMI, and cognitive ability. As a result, scores ranging from 12 to 14, 8–11, and 0–7 represent normal nutrition status, the risk of malnutrition, and malnutrition, respectively. The GDS is often used to detect the depressive symptoms, which comprise of 30 items according to the common topical concerns of the elderly. A score of 0–9 indicates normal or absence of depressive symptoms, 10–30 indicates depression, of which, 10–19 means mild depressive symptoms, whereas 20–30 represents severe depressive symptoms. The MMSE is an instrument to identify the cognitive impairment including 30 questions encompassing 5 aspects of temporal and spatial orientation, memory, attention and calculation, recall ability, and language. Scores of MMSE < 24 indicate cognitive impairment. SPPB is a battery of objective physical performance including three tests of balance, sit-up for five times, and walking speed. The scores range from 0 to 12, and high scores denote superior function of lower extremity [25]. In addition to CGA, demographic information, lifestyle, and clinical profile of participants will be collected based on the gender, age, height, weight, education level, health care type, smoking and alcohol status, chronic comorbidities, echocardiography and laboratory variables.

All subjects who are involved in this study will be categorized into control and intervention groups based on a random number generated by the computer according to the ratio of 1:1 in a blinded manner. The information of random group allocation will be maintained in a sealed envelope opened only by a trial manager who is not involved in the recruitment, assessment, or intervention to assign the treatment. The control group will receive routine diagnosis and treatment, while the intervention group will receive individualized interventions in terms of the consequence of CGA based on routine diagnosis and treatment (Table 2).

**Table 2:** Items of the intervention

Items	Intervention group		Control group
	Methods	Frequency	
Nutrition	Diet recommendation with/without nutritional supplementation	Throughout the trial	Routine treatment
Psychology	Psychotherapy with/without antidepressants	30 min, twice one week	
Physical function	Warm-up	30–45 min, three times one week	
	Walking forwards/backwards		
	Knee and hip extension		
	Static standing on one leg		
	Toe and heel raise		
	Knee lifts		
	Cool-down		

The intervention is not a fixed pattern, which will be performed and adjusted according to the frailty characteristics of participants assessed at baseline

The nutritionist will communicate with all participants to conduct a retrospective investigation on their diet and distribute a handbook to record their daily diet. For reference, the energy and protein/g of common food will be printed in the Handbook (Additional files 1 and 2). The patients of the intervention group with malnutrition or with the risk of malnutrition are recommended an optimal diet structure and daily intake of energy and protein. Nutritional preparations such as homogenate meal, whey protein powder, or enteral nutrition powder will be provided to the intervention group that requires additional supplementation according to the prescription of the nutritionist. Other individuals in the intervention and control groups were administered the same dose of artificial preparation consisting of maltodextrin, sugar, and water. All preparations will be seasoned with vanilla to mask the original flavor and placed in the same opaque bottle. The adherence of participants will be monitored every 2 weeks by phone contact.

The patients with depression, initially evaluated by GDS scale in intervention, would be further assessed by the psychologist and appropriate drugs administered to improve depression. In addition, depressive patients will receive one-to-one psychotherapy twice a week for 30 min each, including supportive psychotherapy, knowledge therapy, and behavioral relaxation therapy. Other enrolled individuals randomly assigned to the control group and assessed as not to be depressed in the intervention group would be guided to treat senescence and diseases in a correct way and be encouraged to socialize and embrace life with a positive attitude. The adherence of participants will be monitored every 2 weeks by phone contact.

The patients of the intervention group with poor physical function would receive exercise prescription tailored to the subjects’ physical ability from a trained specialist. The exercise training of moderate and gradually increasing intensity is home-based, three times a week, 30–45 min/session for 12 months, which aims at improving strength, balance, flexibility, and endurance of the elderly. The exercise will cover walking forwards/backwards, knee and hip extension, static standing on one leg, toe and heel raise, and knee lifts, which based on the literature and the American College of Sports Medicine recommendations for older adults [26, 27]. The emphasis of the training will be adjusted according to the physical performance tests of the participants. As the patient’s physical qualities increases, the training intensity will be gradually increased by increasing the repeated frequency of each action. In order to avoid exercise-related injuries, the corresponding warm-up and cool-down conducted before and after exercise training would be performed. All exercise training will be performed in the patient’s home. The specialist will contact the patients by phone every 2 weeks to monitor the execution of the exercise prescription and resolve the problems raised during training. If necessary, face-to-face guidance will be provided. For participants with normal physical performance in the intervention group and those in the control group, physical activities will be encouraged and a customized exercise program will not be prepared.

Participants with two or more problems will be treated simultaneously by the relevant specialists. Furthermore, the intervention group was subjected to the management of chronic disease and assessment of medication for rational use of medicine, which will be accomplished by geriatric doctors mainly. Discontinuation of the intervention may occur in case of health decline or if a participant wishes to stop taking part in the intervention group. Communication and encouragement will be used to improve adherence to intervention. Reasons and information for drop outs will be documented.

All participants will be followed up for 30 days and 12 months through clinic or telephone contact by specific personnel who are blinded to group allocation to minimize the bias. The content of follow-up will include frailty reassessment using frailty phenotype, frequency, time, main medical conditions of rehospitalization, death and echocardiography.

### Outcomes

Frailty is defined as the primary outcome to evaluate the efficiency of the personalized intervention treatment that reversed frailty to pre-frailty or non-frailty. The secondary outcomes are re-hospitalization and death that assess the effect of frailty on the prognosis of patients, medical examination such as echocardiography.

### Data collection and management

The questionnaires and functional tests will be performed face-to-face, among which, grip strength will be measured by electronic hand dynamometer (Takei Digital Grip Strength Dynamometer, Model T.K.K5401; Takei Scientific Instruments Co., Ltd., Niigata, Japan). Demographic information, concomitant diseases, echocardiography data and laboratory variables of participants will be collected from the data system of Beijing Chaoyang Hospital, Capital Medical University. The frequency, time, and main medical conditions of re-hospitalization will be obtained by telephone contact. All data will be collected by trained study personnel and stored on Microsoft Office Excel under the custody of specific staff. Quality of the entered data will be monitored by checking entry for a random sample of subjects prior to database locking. A data monitoring committee, responsible for data monitoring, interim analyses and auditing.

The study is ongoing. Follow-up visits will complete on September 2020. Statistical analysis and publication of the full results will take place until 2021.

### Statistical analysis

Participants’ demographic information, comorbidities, laboratory results, other clinical characteristics, frailty, consequences of the CGA, and outcomes at 30 days and 12 months will be presented by descriptive statistics. The measurement data in accordance with normal distribution would be described by mean values ± standard deviation, or else by median values and interquartile ranges. The enumeration data would be expressed as percentage or rate. The 95% confidence interval (CI) would be reported subsequently. To compare the differences between means, Student’s t-test will be performed, and the differences between frequencies will be compared by χ^2^ test or Fisher’s exact test. Logistic regression will be conducted to analyze the predictors of frailty and compare the groups with respect to dichotomous outcome variables. The odds ratios will represent the effect of predictors. Kaplan–Meier survival curve would be plotted, and the Cox proportional hazard regression model would assess the association between frailty and mortality. Missing data, due to loss to follow up, will be imputed using the last value carried forwards method [28]. Sensitivity analyses will be conducted to determine whether the results have been influenced by missing data. All data would be analyzed by SPSS version 23 (IBM Corp, Armonk, NY). *P*-values < 0.05 will be considered as statistically significant. All probabilities would be two-tailed.

## Discussion

Frailty is an age-related clinical with a predictive effect on the negative prognosis in older individuals, which might severely affect the quality of life. Strikingly, some studies demonstrated that frailty could be improved or reversed. This phenomenon was confirmed by several randomized controlled trials conducted on different types of individuals. The advantage of the present study is the method of intervention. Unlike a majority of the previous randomized controlled studies, the intervention group in the present study would not be treated in a specific domain such as nutritional, psychological, and physical intervention but with individualized interventions in multiple domains based on the result of CGA used for identifying the patients’ needs. Several studies showed that multi-domain interventions of frailty are effective in improving the frail status of individuals as mentioned above. On the other hand, targeted treatment is strongly recommended as compared to the undifferentiated treatment for precision medicine.

ESRD is an inevitable terminal point for patients with CKD. Owing to economic reasons or considering the quality of life, however, the treatment of ESRD of kidney transplantation or dialysis is not acceptable for a part of patients. The physician can delay the progression of the disease and attempt to improve the quality of life; nevertheless, it is not yet tolerable. A prospective study showed that frailty is associated with increased risk of dialysis therapy, and the conclusion remains unaltered after adjusting for age, sex, BMI, and diabetes [29]. Consequently, ameliorated frailty is beneficial in delaying the progression of CKD in frail patients. Taken together, frailty is simple and easy to assess and the multi-disciplinary intervention is acceptable in clinical practice. If the positive effect of the individualized intervention is demonstrated, this study would provide a new way to treat frail elderly patients undergoing CKD to postpone ESRD.

## Supplementary information

**Additional file 1.** Handbook V1.0.

**Additional file 2.** Handbook (in English).

## Data Availability

Data can be obtained from the corresponding author upon reasonable request.

## Abbreviations

ADL: Activity of daily living
BMI: Body mass index
CGA: Comprehensive geriatric assessment
CI: Confidence interval
CKD: Chronic kidney disease
CKD-EPI: Chronic Kidney Disease Epidemiology Collaboration
eGFR: Estimated glomerular filtration rate
ESRD: End-stage renal disease
GDS: Geriatric depression scale
IADL: Instrumental activity of daily living
MMSE: Mini-mental state examination
MNA-SF: Mini-nutritional assessment short form
SPPB: Short physical performance battery
