# Supplementary material for: Individualized intervention for frail non-dialysis elderly patients with chronic kidney disease: protocol for a randomized controlled trial
Source: BMC Geriatr. 2020 May 4;20:159. doi: 10.1186/s12877-020-1491-6 (PMC7199301; doi:10.1186/s12877-020-1491-6)
Supplement: Supplementary file 1 — Additional file 1. Handbook V1.0. [file 12877_2020_1491_MOESM1_ESM.pdf]

饮食日记

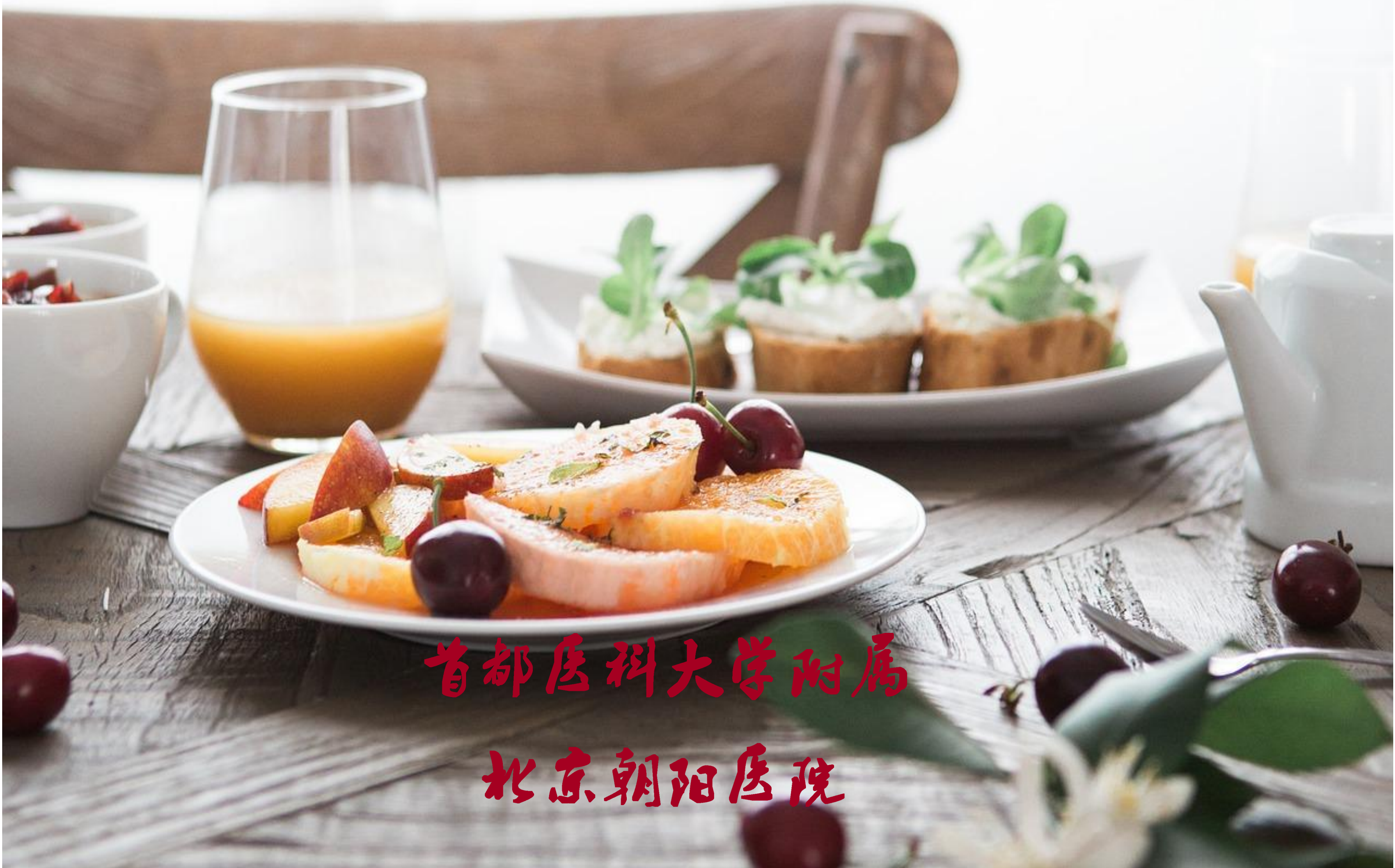

首都医科大学附属

北京朝阳医院

序 言

伴随人口老龄化的快速进展老年人群快速增长，同时老年人群存在异质性特点，人们越来越关注老年人的衰弱问题。衰弱，常常给老年人带来不良的临床预后，如住院、病死率增高等。在大多数老年衰弱患者中，存在营养状况不佳和营养需求变化。因此，营养支持及日常健康饮食对衰弱患者非常重要。

这个《饮食日记》是一本简单而有效的手册，用于帮助您掌握自己的日常饮食。您可以记录您的日常饮食，这将对您日常饮食中摄入的热量及蛋白质有直观的认识。在这个手册的后面部分，附上了关于常见食物的热量（卡路里）及蛋白质含量对照表，可以作为日常饮食的参考。

这本手册使用起来很方便，只需要您记录下来日期及您进食的食物，包括食物名称及量，进而可参照后边的食物附录，换算出您摄入的卡路里及蛋白质含量。

如果您有任何问题，可与我们专业的工作人员联系：010-85231436。

孙倩美

首都医科大学附属北京朝阳医院综合科

2017 年 9 月

姓名___________

日期___________

	食物	总量 (g)	可食用部分 (g)	卡路里 (kcal)	蛋白质 (g)
早餐					
加餐 1					
午餐					
加餐 2					
晚餐					
加餐 3					
合计	-		-		

姓名__________

日期__________

	食物	总量 (g)	可食用部分 (g)	卡路里 (kcal)	蛋白质 (g)
早餐					
加餐 1					
午餐					
加餐 2					
晚餐					
加餐 3					
合计	-		-		

姓名__________

日期__________

	食物	总量 (g)	可食用部分 (g)	卡路里 (kcal)	蛋白质 (g)
早餐					
加餐 1					
午餐					
加餐 2					
晚餐					
加餐 3					
合计	-		-		

姓名___________

日期___________

	食物	总量 (g)	可食用部分 (g)	卡路里 (kcal)	蛋白质 (g)
早餐					
加餐 1					
午餐					
加餐 2					
晚餐					
加餐 3					
合计	-		-		

姓名__________

日期__________

	食物	总量 (g)	可食用部分 (g)	卡路里 (kcal)	蛋白质 (g)
早餐					
加餐 1					
午餐					
加餐 2					
晚餐					
加餐 3					
合计	-		-		

姓名__________

日期__________

	食物	总量<br>(g)	可食用部分<br>(g)	卡路里<br>(kcal)	蛋白质<br>(g)
早餐					
加餐 1					
午餐					
加餐 2					
晚餐					
加餐 3					
合计	-		-		

姓名__________

日期__________

	食物	总量 (g)	可食用部分 (g)	卡路里 (kcal)	蛋白质 (g)
早餐					
加餐 1					
午餐					
加餐 2					
晚餐					
加餐 3					
合计	-		-		

姓名__________

日期__________

	食物	总量 (g)	可食用部分 (g)	卡路里 (kcal)	蛋白质 (g)
早餐					
加餐 1					
午餐					
加餐 2					
晚餐					
加餐 3					
合计	-		-		

姓名__________

日期__________

	食物	总量 (g)	可食用部分 (g)	卡路里 (kcal)	蛋白质 (g)
早餐					
加餐 1					
午餐					
加餐 2					
晚餐					
加餐 3					
合计	-		-		

姓名__________

日期__________

	食物	总量 (g)	可食用部分 (g)	卡路里 (kcal)	蛋白质 (g)
早餐					
加餐 1					
午餐					
加餐 2					
晚餐					
加餐 3					
合计	-		-		

姓名__________

日期__________

	食物	总量 (g)	可食用部分 (g)	卡路里 (kcal)	蛋白质 (g)
早餐					
加餐 1					
午餐					
加餐 2					
晚餐					
加餐 3					
合计	-		-		

姓名__________

日期__________

	食物	总量(g)	可食用部分(g)	卡路里(kcal)	蛋白质(g)
早餐					
加餐 1					
午餐					
加餐 2					
晚餐					
加餐 3					
合计	-		-		

姓名__________

日期__________

	食物	总量 (g)	可食用部分 (g)	卡路里 (kcal)	蛋白质 (g)
早餐					
加餐 1					
午餐					
加餐 2					
晚餐					
加餐 3					
合计	-		-		

姓名__________

日期__________

	食物	总量 (g)	可食用部分 (g)	卡路里 (kcal)	蛋白质 (g)
早餐					
加餐 1					
午餐					
加餐 2					
晚餐					
加餐 3					
合计	-		-		

姓名__________

日期__________

	食物	总量<br>(g)	可食用部分<br>(g)	卡路里<br>(kcal)	蛋白质<br>(g)
早餐					
加餐 1					
午餐					
加餐 2					
晚餐					
加餐 3					
合计	-		-		

姓名___________

日期___________

	食物	总量 (g)	可食用部分 (g)	卡路里 (kcal)	蛋白质 (g)
早餐					
加餐 1					
午餐					
加餐 2					
晚餐					
加餐 3					
合计	-		-		

姓名__________

日期__________

	食物	总量 (g)	可食用部分 (g)	卡路里 (kcal)	蛋白质 (g)
早餐					
加餐 1					
午餐					
加餐 2					
晚餐					
加餐 3					
合计	-		-		

姓名__________

日期__________

	食物	总量 (g)	可食用部分 (g)	卡路里 (kcal)	蛋白质 (g)
早餐					
加餐 1					
午餐					
加餐 2					
晚餐					
加餐 3					
合计	-		-		

姓名___________

日期___________

	食物	总量 (g)	可食用部分 (g)	卡路里 (kcal)	蛋白质 (g)
早餐					
加餐 1					
午餐					
加餐 2					
晚餐					
加餐 3					
合计	-		-		

姓名__________

日期__________

	食物	总量 (g)	可食用部分 (g)	卡路里 (kcal)	蛋白质 (g)
早餐					
加餐 1					
午餐					
加餐 2					
晚餐					
加餐 3					
合计	-		-		

姓名___________

日期___________

	食物	总量 (g)	可食用部分 (g)	卡路里 (kcal)	蛋白质 (g)
早餐					
加餐 1					
午餐					
加餐 2					
晚餐					
加餐 3					
合计	-		-		

姓名__________

日期__________

	食物	总量(g)	可食用部分(g)	卡路里(kcal)	蛋白质(g)
早餐					
加餐 1					
午餐					
加餐 2					
晚餐					
加餐 3					
合计	-		-		

姓名__________

日期__________

	食物	总量 (g)	可食用部分 (g)	卡路里 (kcal)	蛋白质 (g)
早餐					
加餐 1					
午餐					
加餐 2					
晚餐					
加餐 3					
合计	-		-		

姓名__________

日期__________

	食物	总量 (g)	可食用部分 (g)	卡路里 (kcal)	蛋白质 (g)
早餐					
加餐 1					
午餐					
加餐 2					
晚餐					
加餐 3					
合计	-		-		

姓名__________

日期__________

	食物	总量 (g)	可食用部分 (g)	卡路里 (kcal)	蛋白质 (g)
早餐					
加餐 1					
午餐					
加餐 2					
晚餐					
加餐 3					
合计	-		-		

姓名__________

日期__________

	食物	总量 (g)	可食用部分 (g)	卡路里 (kcal)	蛋白质 (g)
早餐					
加餐 1					
午餐					
加餐 2					
晚餐					
加餐 3					
合计	-		-		

姓名__________

日期__________

	食物	总量 (g)	可食用部分 (g)	卡路里 (kcal)	蛋白质 (g)
早餐					
加餐 1					
午餐					
加餐 2					
晚餐					
加餐 3					
合计	-		-		

姓名__________

日期__________

	食物	总量 (g)	可食用部分 (g)	卡路里 (kcal)	蛋白质 (g)
早餐					
加餐 1					
午餐					
加餐 2					
晚餐					
加餐 3					
合计	-		-		

姓名__________

日期__________

	食物	总量 (g)	可食用部分 (g)	卡路里 (kcal)	蛋白质 (g)
早餐					
加餐 1					
午餐					
加餐 2					
晚餐					
加餐 3					
合计	-		-		

姓名__________

日期__________

	食物	总量 (g)	可食用部分 (g)	卡路里 (kcal)	蛋白质 (g)
早餐					
加餐 1					
午餐					
加餐 2					
晚餐					
加餐 3					
合计	-		-		

常见食物附录

食物名称	食物总量 (g)	可食用部分 (g)	卡路里 (kcal)	蛋白质 (g)	脂肪 (g)	膳食纤维 (g)	碳水化合物 (g)
稻米	100	100	**345**	**7.7**	0.6	0.6	77.4
糯米	100	100	**344**	**7.9**	0.8	0.7	76.7
小麦	100	100	**339**	**11.9**	-	10.8	75.2
小麦粉	100	100	**349**	**11.2**	1.5	2.1	73.6
挂面	100	100	**348**	**10.1**	0.7	1.6	76.0
荞麦	100	100	**337**	**9.3**	2.3	6.5	73.0
大麦	100	100	**327**	**10.2**	1.4	9.9	73.3
小米	100	100	**361**	**9.0**	3.1	1.6	75.1
玉米	100	100	**348**	**8.7**	3.8	6.4	73.0
玉米面	100	100	**352**	**8.1**	3.3	5.6	75.2
燕麦片	100	100	**367**	**15.0**	6.7	5.3	61.6
方便面	100	100	**472**	**9.5**	21.1	0.7	60.9
面包	100	100	**313**	**8.3**	5.1	0.5	58.6
红薯	100	100	**61**	**0.7**	0.2	0.6	15.3
土豆	100	100	**81**	**2.6**	0.2	1.1	17.8
包子	100	100	**227**	**7.3**	10.0	1.70	30.3
饺子	100	100	**218**	**7.5**	10.8	3.2	26.0
年糕	100	100	**156**	**3.3**	0.6	0.8	34.7
炒饭	100	100	**188**	**5.0**	5.6	2.0	31.7
牛奶	100	100	**54**	**3.0**	3.2	-	3.4
酸奶	100	100	**72**	**2.5**	2.7	-	9.3
三明治	100	100	**244**	**14.2**	10.6	1.0	22.9
奶粉	100	100	**478**	**20.1**	21.2	-	51.7
奶酪	100	100	**328**	**25.7**	23.5	-	3.5
卷心菜	100	82	**26**	**2.1**	0.2	1.2	4.6
大白菜	100	83	**17**	**1.4**	0.1	0.9	3.0
油菜绿菜	100	87	**25**	**1.8**	0.5	1.1	3.8
芹菜	100	66	**17**	**0.8**	0.1	1.4	3.9
韭菜	100	90	**29**	**2.4**	0.4	1.4	4.6
芦笋	100	90	**22**	**1.4**	0.1	1.9	4.9

食物名称	食物总量 (g)	可食用部分 (g)	卡路里 (kcal)	蛋白质 (g)	脂肪 (g)	膳食纤维 (g)	碳水化合物 (g)
莴苣	100	62	**15**	**1.0**	0.1	0.6	2.8
大蒜	100	85	**128**	**4.5**	0.2	1.1	27.6
茭白	100	76	**26**	**1.2**	0.2	1.9	5.9
胡萝卜	100	96	**39**	**1.0**	0.2	1.1	8.8
荸荠	100	78	**61**	**1.2**	0.2	1.1	14.2
姜	100	95	**46**	**1.3**	0.6	2.7	10.3
萝卜	100	95	**23**	**0.9**	0.1	1.0	5.0
马铃薯	100	94	**77**	**2.0**	0.2	0.7	17.2
藕	100	88	**73**	**1.9**	0.2	1.2	1.0
苹果	100	76	**54**	**0.2**	0.2	1.2	13.5
葡萄	100	86	**44**	**0.5**	0.2	0.4	10.3
香蕉	100	59	**93**	**1.4**	0.2	1.2	22.0
草莓	100	97	**32**	**1.0**	0.2	1.1	7.1
桂圆	100	50	**71**	**1.2**	0.1	0.4	16.6
红果	100	100	**251**	**4.3**	2.2	49.7	78.4
梨	100	82	**45**	**0.2**	0.2	1.1	11.1
枇杷	100	62	**41**	**0.8**	0.2	0.8	9.3
菠萝	100	68	**44**	**0.5**	0.1	1.3	10.8
鲜枣	100	87	**125**	**0.3**	1.1	1.9	30.5
柿	100	87	**74**	**0.4**	0.1	1.4	18.5
桃	100	86	**51**	**0.9**	0.1	1.3	12.2
杏	100	91	**38**	**0.9**	0.1	1.3	9.1
橘	100	76	**45**	**0.8**	0.4	1.4	10.3
猪肉(瘦)	100	100	**143**	**20.3**	6.2	-	1.5
羊肉(瘦)	100	90	**118**	**20.5**	3.9	-	0.2
牛肉(瘦)	100	100	**106**	**20.2**	2.3	-	1.2
酱牛肉	100	100	**246**	**31.4**	11.9	-	3.2
鸡	100	66	**167**	**19.3**	9.4	-	1.3
鹅	100	63	**251**	**17.9**	19.9	-	-
鸽	100	42	**201**	**16.5**	14.2	-	1.7
鸭	100	68	**240**	**15.5**	19.7	-	0.2
北京烤鸭	100	80	**436**	**16.6**	38.4	-	6.0
大黄鱼	100	66	**97**	**17.7**	2.5	-	0.8
小黄鱼	100	63	**99**	**17.9**	3.0	-	0.1
带鱼	100	76	**127**	**17.7**	4.9	-	3.1
鲢鱼	100	61	**104**	**17.8**	3.6	-	-
鲤鱼	100	54	**109**	**17.6**	4.1	-	0.5

食物名称	食物总量 (g)	可食用部分 (g)	卡路里 (kcal)	蛋白质 (g)	脂肪 (g)	膳食纤维 (g)	碳水化合物 (g)
鳝鱼	100	67	**89**	**18.0**	1.4	-	1.2
草鱼	100	58	**113**	**16.6**	5.2	-	-
泥鳅	100	60	**96**	**17.9**	2.0	-	1.7
墨鱼	100	69	**83**	**15.2**	0.9	-	3.4
对虾	100	61	**93**	**18.6**	0.8	-	2.8
河虾	100	86	**87**	**16.4**	2.4	-	-
螃蟹	100	42	**103**	**17.5**	2.6	-	2.3
鸡蛋	100	88	**156**	**12.8**	11.1	-	1.3
鸭蛋	100	87	**180**	**12.6**	13.0	-	3.1
鹅蛋	100	87	**196**	**11.1**	15.6	-	2.8
鹌鹑蛋	100	86	**160**	**12.8**	11.1	-	2.1
核桃	100	43	**646**	**14.9**	58.8	9.5	19.1
花生	100	100	**574**	**24.8**	44.3	5.5	21.7
莲子	100	100	**350**	**17.2**	2.0	3.0	67.2
山核桃	100	24	**616**	**18.0**	50.4	7.4	26.2
西瓜子	100	43	**582**	**32.7**	44.8	4.5	14.2

参考文献：

1. 季兰芳，等. 营养与膳食[M]. 第三版. 北京：人民卫生出版社，2014.

2. 焦广宇，等. 临床营养学[M]. 第三版. 北京：人民卫生出版社，2010.
