# Supplementary material for: Individualized intervention for frail non-dialysis elderly patients with chronic kidney disease: protocol for a randomized controlled trial
Source: BMC Geriatr. 2020 May 4;20:159. doi: 10.1186/s12877-020-1491-6 (PMC7199301; doi:10.1186/s12877-020-1491-6)
Supplement: Supplementary file 2 — Additional file 2. Handbook (in English). [file 12877_2020_1491_MOESM2_ESM.pdf]

DIET DIARY

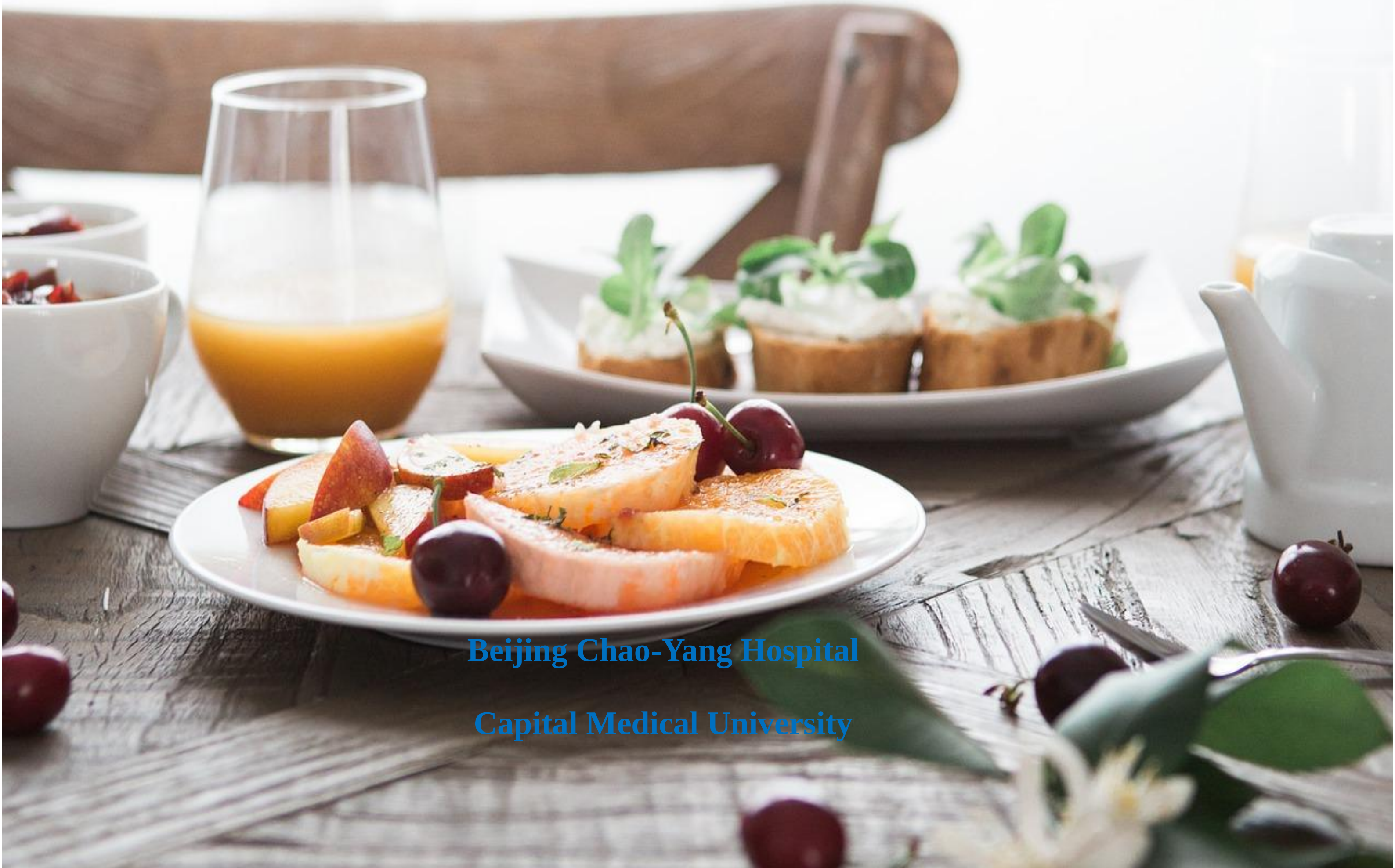

Beijing Chao-Yang Hospital

Capital Medical University

INTRODUCTION

The accelerated speed of aging and the heterogeneity of elderly have led to an increasing focus on the frailty. And, frailty is consistently believed to be associated with hospitalization, mortality, and other poor outcomes. In most of frail patients, nutrition worsened and nutritional requirements are altered. So, nutrition support and rational diet are very important for frail patients.

This diet diary is a simple but helpful manual which can help you manage your diet. You can record your daily diet and gain a straightforward impression of how calories and proteins you get in your day. In the last part of the handbook, we provide an appendix for calories and proteins of common foods for reference.

The way of using the booklet is easy, which only needs you to put down the date and the food you eat on that day, including the name and amount, and then calculate the total calories and proteins intakes for the day according to the appendix.

If you have any problem in the process of using, please contact the number 010-85231436.

Qian-Mei Sun
Department of Internal Medicine,
Beijing Chao-Yang Hospital,
Capital Medical University, Beijing, China
September, 2017

NAME___________

DATE____________

	Foods	Amount (g)	Edible part (g)	Calories (kcal)	Proteins (g)
**Breakfast**					
**Additional intake 1**					
**Lunch**					
**Additional intake 2**					
**Dinner**					
**Additional intake 3**					
**Total**	-		-		

NAME___________

DATE____________

	Foods	Amount (g)	Edible part (g)	Calories (kcal)	Proteins (g)
**Breakfast**					
**Additional intake 1**					
**Lunch**					
**Additional intake 2**					
**Dinner**					
**Additional intake 3**					
**Total**	-		-		

NAME__________

DATE___________

	Foods	Amount (g)	Edible part (g)	Calories (kcal)	Proteins (g)
**Breakfast**					
**Additional intake 1**					
**Lunch**					
**Additional intake 2**					
**Dinner**					
**Additional intake 3**					
**Total**	-		-		

NAME___________

DATE____________

	Foods	Amount (g)	Edible part (g)	Calories (kcal)	Proteins (g)
**Breakfast**					
**Additional intake 1**					
**Lunch**					
**Additional intake 2**					
**Dinner**					
**Additional intake 3**					
**Total**	-		-		

NAME___________

DATE____________

	Foods	Amount (g)	Edible part (g)	Calories (kcal)	Proteins (g)
**Breakfast**					
**Additional intake 1**					
**Lunch**					
**Additional intake 2**					
**Dinner**					
**Additional intake 3**					
**Total**	-		-		

NAME__________

DATE___________

	Foods	Amount (g)	Edible part (g)	Calories (kcal)	Proteins (g)
**Breakfast**					
**Additional intake 1**					
**Lunch**					
**Additional intake 2**					
**Dinner**					
**Additional intake 3**					
**Total**	-		-		

NAME__________

DATE___________

	Foods	Amount (g)	Edible part (g)	Calories (kcal)	Proteins (g)
**Breakfast**					
**Additional intake 1**					
**Lunch**					
**Additional intake 2**					
**Dinner**					
**Additional intake 3**					
**Total**	-		-		

北京朝阳医院
BEIJING CHAO-YANG HOSPITAL

NAME___________

DATE____________

	Foods	Amount (g)	Edible part (g)	Calories (kcal)	Proteins (g)
**Breakfast**					
**Additional intake 1**					
**Lunch**					
**Additional intake 2**					
**Dinner**					
**Additional intake 3**					
**Total**	-		-		

NAME__________

DATE___________

	Foods	Amount (g)	Edible part (g)	Calories (kcal)	Proteins (g)
**Breakfast**					
**Additional intake 1**					
**Lunch**					
**Additional intake 2**					
**Dinner**					
**Additional intake 3**					
**Total**	-		-		

NAME__________

DATE___________

	Foods	Amount (g)	Edible part (g)	Calories (kcal)	Proteins (g)
**Breakfast**					
**Additional intake 1**					
**Lunch**					
**Additional intake 2**					
**Dinner**					
**Additional intake 3**					
**Total**	-		-		

NAME__________

DATE___________

	Foods	Amount (g)	Edible part (g)	Calories (kcal)	Proteins (g)
**Breakfast**					
**Additional intake 1**					
**Lunch**					
**Additional intake 2**					
**Dinner**					
**Additional intake 3**					
**Total**	-		-		

北京朝阳医院
BEIJING CHAO-YANG HOSPITAL

NAME__________

DATE___________

	Foods	Amount (g)	Edible part (g)	Calories (kcal)	Proteins (g)
**Breakfast**					
**Additional intake 1**					
**Lunch**					
**Additional intake 2**					
**Dinner**					
**Additional intake 3**					
**Total**	-		-		

NAME___________

DATE____________

	Foods	Amount (g)	Edible part (g)	Calories (kcal)	Proteins (g)
**Breakfast**					
**Additional intake 1**					
**Lunch**					
**Additional intake 2**					
**Dinner**					
**Additional intake 3**					
**Total**	-		-		

NAME___________

DATE____________

	Foods	Amount (g)	Edible part (g)	Calories (kcal)	Proteins (g)
**Breakfast**					
**Additional intake 1**					
**Lunch**					
**Additional intake 2**					
**Dinner**					
**Additional intake 3**					
**Total**	-		-		

NAME__________

DATE___________

	Foods	Amount (g)	Edible part (g)	Calories (kcal)	Proteins (g)
**Breakfast**					
**Additional intake 1**					
**Lunch**					
**Additional intake 2**					
**Dinner**					
**Additional intake 3**					
**Total**	-		-		

NAME__________

DATE___________

	Foods	Amount (g)	Edible part (g)	Calories (kcal)	Proteins (g)
**Breakfast**					
**Additional intake 1**					
**Lunch**					
**Additional intake 2**					
**Dinner**					
**Additional intake 3**					
**Total**	-		-		

NAME__________

DATE___________

	Foods	Amount (g)	Edible part (g)	Calories (kcal)	Proteins (g)
**Breakfast**					
**Additional intake 1**					
**Lunch**					
**Additional intake 2**					
**Dinner**					
**Additional intake 3**					
**Total**	-		-		

北京朝阳医院
BEIJING CHAO-YANG HOSPITAL

NAME___________

DATE____________

	Foods	Amount (g)	Edible part (g)	Calories (kcal)	Proteins (g)
**Breakfast**					
**Additional intake 1**					
**Lunch**					
**Additional intake 2**					
**Dinner**					
**Additional intake 3**					
**Total**	-		-		

NAME__________

DATE___________

	Foods	Amount (g)	Edible part (g)	Calories (kcal)	Proteins (g)
**Breakfast**					
**Additional intake 1**					
**Lunch**					
**Additional intake 2**					
**Dinner**					
**Additional intake 3**					
**Total**	-		-		

北京朝阳医院
BEIJING CHAO-YANG HOSPITAL

NAME__________

DATE___________

	Foods	Amount (g)	Edible part (g)	Calories (kcal)	Proteins (g)
**Breakfast**					
**Additional intake 1**					
**Lunch**					
**Additional intake 2**					
**Dinner**					
**Additional intake 3**					
**Total**	-		-		

NAME__________

DATE___________

	Foods	Amount (g)	Edible part (g)	Calories (kcal)	Proteins (g)
**Breakfast**					
**Additional intake 1**					
**Lunch**					
**Additional intake 2**					
**Dinner**					
**Additional intake 3**					
**Total**	-		-		

NAME___________

DATE____________

	Foods	Amount (g)	Edible part (g)	Calories (kcal)	Proteins (g)
**Breakfast**					
**Additional intake 1**					
**Lunch**					
**Additional intake 2**					
**Dinner**					
**Additional intake 3**					
**Total**	-		-		

NAME__________

DATE___________

	Foods	Amount (g)	Edible part (g)	Calories (kcal)	Proteins (g)
**Breakfast**					
**Additional intake 1**					
**Lunch**					
**Additional intake 2**					
**Dinner**					
**Additional intake 3**					
**Total**	-		-		

北京朝阳医院
BEIJING CHAO-YANG HOSPITAL

NAME__________

DATE___________

	Foods	Amount (g)	Edible part (g)	Calories (kcal)	Proteins (g)
**Breakfast**					
**Additional intake 1**					
**Lunch**					
**Additional intake 2**					
**Dinner**					
**Additional intake 3**					
**Total**	-		-		

NAME___________

DATE____________

	Foods	Amount (g)	Edible part (g)	Calories (kcal)	Proteins (g)
**Breakfast**					
**Additional intake 1**					
**Lunch**					
**Additional intake 2**					
**Dinner**					
**Additional intake 3**					
**Total**	-		-		

北京朝阳医院
BEIJING CHAO-YANG HOSPITAL

NAME__________

DATE___________

	Foods	Amount (g)	Edible part (g)	Calories (kcal)	Proteins (g)
**Breakfast**					
**Additional intake 1**					
**Lunch**					
**Additional intake 2**					
**Dinner**					
**Additional intake 3**					
**Total**	-		-		

NAME___________

DATE____________

	Foods	Amount (g)	Edible part (g)	Calories (kcal)	Proteins (g)
**Breakfast**					
**Additional intake 1**					
**Lunch**					
**Additional intake 2**					
**Dinner**					
**Additional intake 3**					
**Total**	-		-		

NAME__________

DATE___________

	Foods	Amount (g)	Edible part (g)	Calories (kcal)	Proteins (g)
**Breakfast**					
**Additional intake 1**					
**Lunch**					
**Additional intake 2**					
**Dinner**					
**Additional intake 3**					
**Total**	-		-		

NAME___________

DATE____________

	Foods	Amount (g)	Edible part (g)	Calories (kcal)	Proteins (g)
**Breakfast**					
**Additional intake 1**					
**Lunch**					
**Additional intake 2**					
**Dinner**					
**Additional intake 3**					
**Total**	-		-		

北京朝阳医院 BEIJING CHAO-YANG HOSPITAL

NAME__________

DATE___________

	Foods	Amount (g)	Edible part (g)	Calories (kcal)	Proteins (g)
**Breakfast**					
**Additional intake 1**					
**Lunch**					
**Additional intake 2**					
**Dinner**					
**Additional intake 3**					
**Total**	-		-		

APPENDIX

Food name	Amount (g)	Edible part (g)	Calories (kcal)	Proteins (g)	Fat (g)	Dietary fiber (g)	Carbohydrate (g)
**Rice**	100	100	**345**	**7.7**	0.6	0.6	77.4
**Glutinous rice**	100	100	**344**	**7.9**	0.8	0.7	76.7
**Wheat**	100	100	**339**	**11.9**	-	10.8	75.2
**Wheatmeal**	100	100	**349**	**11.2**	1.5	2.1	73.6
**Noodles**	100	100	**348**	**10.1**	0.7	1.6	76.0
**Buckwheat**	100	100	**337**	**9.3**	2.3	6.5	73.0
**Barley**	100	100	**327**	**10.2**	1.4	9.9	73.3
**Millet**	100	100	**361**	**9.0**	3.1	1.6	75.1
**Corn**	100	100	**348**	**8.7**	3.8	6.4	73.0
**Corn meal**	100	100	**352**	**8.1**	3.3	5.6	75.2
**Oats**	100	100	**367**	**15.0**	6.7	5.3	61.6
**Instant noodles**	100	100	**472**	**9.5**	21.1	0.7	60.9
**Bread**	100	100	**313**	**8.3**	5.1	0.5	58.6
**Sweet potato**	100	100	**61**	**0.7**	0.2	0.6	15.3
**Potato**	100	100	**81**	**2.6**	0.2	1.1	17.8
**Stuffed bun**	100	100	**227**	**7.3**	10.0	1.70	30.3
**Dumplings**	100	100	**218**	**7.5**	10.8	3.2	26.0
**Rice cake**	100	100	**156**	**3.3**	0.6	0.8	34.7
**Fried rice**	100	100	**188**	**5.0**	5.6	2.0	31.7
**Milk**	100	100	**54**	**3.0**	3.2	-	3.4
**Yogurt**	100	100	**72**	**2.5**	2.7	-	9.3
**Sandwich**	100	100	**244**	**14.2**	10.6	1.0	22.9
**Milk powder**	100	100	**478**	**20.1**	21.2	-	51.7
**Cheese**	100	100	**328**	**25.7**	23.5	-	3.5
**Cabbage**	100	82	**26**	**2.1**	0.2	1.2	4.6
**Chinese cabbage**	100	83	**17**	**1.4**	0.1	0.9	3.0
**Green vegetables**	100	87	**25**	**1.8**	0.5	1.1	3.8
**Celery**	100	66	**17**	**0.8**	0.1	1.4	3.9
**Leek**	100	90	**29**	**2.4**	0.4	1.4	4.6
**Asparagus**	100	90	**22**	**1.4**	0.1	1.9	4.9

Food name	Amount (g)	Edible part (g)	Calories (kcal)	Proteins (g)	Fat (g)	Dietary fiber (g)	Carbohydrate (g)
Lettuce	100	62	15	1.0	0.1	0.6	2.8
Garlic	100	85	128	4.5	0.2	1.1	27.6
Water bamboo	100	76	26	1.2	0.2	1.9	5.9
Carrot	100	96	39	1.0	0.2	1.1	8.8
Water chestnut	100	78	61	1.2	0.2	1.1	14.2
Ginger	100	95	46	1.3	0.6	2.7	10.3
Radish	100	95	23	0.9	0.1	1.0	5.0
Potato	100	94	77	2.0	0.2	0.7	17.2
Lotus root	100	88	73	1.9	0.2	1.2	1.0
Apple	100	76	54	0.2	0.2	1.2	13.5
Grapes	100	86	44	0.5	0.2	0.4	10.3
Banana	100	59	93	1.4	0.2	1.2	22.0
Strawberry	100	97	32	1.0	0.2	1.1	7.1
Longan	100	50	71	1.2	0.1	0.4	16.6
Crabapple	100	100	251	4.3	2.2	49.7	78.4
Pear	100	82	45	0.2	0.2	1.1	11.1
Loquat	100	62	41	0.8	0.2	0.8	9.3
Pineapple	100	68	44	0.5	0.1	1.3	10.8
Fresh jujube	100	87	125	0.3	1.1	1.9	30.5
Persimmon	100	87	74	0.4	0.1	1.4	18.5
Peach	100	86	51	0.9	0.1	1.3	12.2
Apricot	100	91	38	0.9	0.1	1.3	9.1
Orange	100	76	45	0.8	0.4	1.4	10.3
Pork (thin)	100	100	143	20.3	6.2	-	1.5
Lamb (thin)	100	90	118	20.5	3.9	-	0.2
Beef (thin)	100	100	106	20.2	2.3	-	1.2
Spiced beef	100	100	246	31.4	11.9	-	3.2
Chicken	100	66	167	19.3	9.4	-	1.3
Goose	100	63	251	17.9	19.9	-	-
Pigeon	100	42	201	16.5	14.2	-	1.7
Duck	100	68	240	15.5	19.7	-	0.2
Roast Duck	100	80	436	16.6	38.4	-	6.0
Pseudosciaena crocea	100	66	97	17.7	2.5	-	0.8
Little croaker	100	63	99	17.9	3.0	-	0.1
Hairtail	100	76	127	17.7	4.9	-	3.1
Chub	100	61	104	17.8	3.6	-	-
Carp	100	54	109	17.6	4.1	-	0.5

Food name	Amount (g)	Edible part (g)	Calories (kcal)	Proteins (g)	Fat (g)	Dietary fiber (g)	Carbohydrate (g)
**Eel**	100	67	**89**	**18.0**	1.4	-	1.2
**Grass carp**	100	58	**113**	**16.6**	5.2	-	-
**Loach**	100	60	**96**	**17.9**	2.0	-	1.7
**Cuttlefish**	100	69	**83**	**15.2**	0.9	-	3.4
**Prawn**	100	61	**93**	**18.6**	0.8	-	2.8
**Shrimp**	100	86	**87**	**16.4**	2.4	-	-
**Crab**	100	42	**103**	**17.5**	2.6	-	2.3
**Egg**	100	88	**156**	**12.8**	11.1	-	1.3
**Duck's egg**	100	87	**180**	**12.6**	13.0	-	3.1
**Goose's egg**	100	87	**196**	**11.1**	15.6	-	2.8
**Quail egg**	100	86	**160**	**12.8**	11.1	-	2.1
**Juglans regia**	100	43	**646**	**14.9**	58.8	9.5	19.1
**Peanut**	100	100	**574**	**24.8**	44.3	5.5	21.7
**Lotus seed**	100	100	**350**	**17.2**	2.0	3.0	67.2
**Pecan**	100	24	**616**	**18.0**	50.4	7.4	26.2
**Water melonseed**	100	43	**582**	**32.7**	44.8	4.5	14.2

Reference:

1. Lan-Fang Ji, etal. Nutrition and Diet [M]. 3rd. Beijing: People's Medical Publishing House (PMPH), 2014.

2. Guang-Yu Jiao, etal. Clinical Nutriology[M]. 3rd. Beijing: People's Medical Publishing House (PMPH), 2010.
